# Older soft tissue sarcoma patients experience increased rates of venous thromboembolic events: a retrospective cohort study of SEER-Medicare data

**DOI:** 10.1186/s13569-015-0033-z

**Published:** 2015-07-26

**Authors:** Sumitra Shantakumar, Alexandra Connelly-Frost, Monica G Kobayashi, Robert Allis, Li Li

**Affiliations:** Worldwide Epidemiology, Research and Development, GlaxoSmithKline, 150 Beach Road, #26-00 Gateway West, Singapore, 189720 Singapore; Frost Consulting, Epidemiologic Research and Grant Writing, Charlotte, NC USA; Worldwide Epidemiology, Research and Development, GlaxoSmithKline, Research Triangle Park, NC USA; Center for Observational Data Analytics, Amgen, Thousand Oaks, CA USA

**Keywords:** Soft tissue sarcoma, Venous thromboembolic events, Deep vein thrombosis, Pulmonary embolism, Co-morbidity, Elderly

## Abstract

**Background:**

Venous thromboembolic co-morbidities can have a significant impact on treatment response, treatment options, quality of life, and ultimately, survival from cancer. There is a dearth of published information on venous thromboembolic co-morbidity among older soft tissue sarcoma patients.

**Methods:**

SEER-Medicare linked data (1993–2005) was utilized for this retrospective cohort analysis (n = 3,480 soft tissue sarcoma patients). Non-cancer patients were frequency-matched by age to cancer patients at a ratio of 1:1; coverage and follow-up requirements were the same as for soft tissue sarcoma cases. Venous thromboembolic events were divided into three groups of interest: deep vein thrombosis, pulmonary embolism, and other thromboembolic events. Relative incidence rates of venous thromboembolic events in soft tissue sarcoma patients with a recent history of cardiovascular event or venous thromboembolic event (12 months before diagnosis) versus soft tissue sarcoma patients without such a recent history were calculated using the Cox proportional hazard models. The Cox proportional hazard model was used to build predictive models to identify important risk factors for each venous thromboembolic event of interest among soft tissue sarcoma patients. Relative incidence rate of VTEs in cancer patients (12 months after diagnosis) versus non-cancer cases (12 months after index date) was calculated using multivariable Cox proportional hazard models.

**Results:**

We observed that among older soft tissue sarcoma patients, 10.6% experienced a deep vein thrombosis, 3.0% experienced a pulmonary embolism, and 3.1% experienced other thromboembolic events in the 12 months after sarcoma diagnosis. On average, 60% of venous thromboembolic events occurred in the first 90 days after sarcoma diagnosis. The highest rates of deep vein thrombosis and pulmonary embolism after sarcoma diagnosis were seen in patients with sarcoma not otherwise specified (deep vein thrombosis: 204/1,000 p-y and pulmonary embolism: 50/1,000 p-y). Recent history of a venous thromboembolic event was the strongest predictor of a subsequent venous thromboembolic event after soft tissue sarcoma diagnosis.

**Conclusion:**

Venous thromboembolic events are common and serious co-morbidities that should be closely monitored in older soft tissue sarcoma patients.

## Background

Venous thromboembolic co-morbidities among cancer patients can have a significant impact on quality of life, treatment options, treatment response, and ultimately, survival from cancer [1–3]. A small body of evidence is developing for brain, breast, lung, ovarian, and pancreatic cancers, suggesting that the incidence of venous thromboembolic events (VTEs) VTEs varies substantially by cancer subtype [2, 4–13]. Estimates for the occurrence of VTEs in these cancers range from as low as 0.4% up to 26.0%, depending on the cancer type, study population, and the length of follow-up [12]. Because the risk of VTEs differs by the histological type of each cancer, it is important to carefully characterize this co-morbidity by cancer subtype.

There is a dearth of information on the extent of venous thromboembolic co-morbidity among older soft tissue sarcoma (STS) patients in the literature. VTE incidence among STS patients has rarely been addressed, timing of VTEs has not been investigated, co-factors have not been considered, soft tissue and bone sarcomas are usually combined, and older populations have not been studied. It is important to understand the scope of VTEs, before and after diagnosis, in order to offer STS patients optimal care and improved quality of life.

The main goals of this study were (1) to estimate the incidence of VTEs before STS diagnosis and during 90-day time periods after STS diagnosis and (2) to produce adjusted relative risk estimates of VTEs for STS patients with and without a cardiovascular disease or VTE history and (3) to compare risk of VTEs for STS patients versus age-matched non-cancer individuals.

## Methods

### Study population

SEER-Medicare data is a linkage of US cancer registry data with Medicare claims data. This database combines two large, population-based, geographically diverse US data sources, providing detailed information about older persons (≥65 years) with and without cancer. Data from 1993 to 2005 were utilized for this retrospective cohort analysis. Patients 65 years of age and over who were diagnosed with STS and had at least 24 months of continuous non-HMO Medicare coverage (Parts A and B) before diagnosis and 1–12 months of follow-up information after diagnosis were included in the cancer cohort. Duration of patient follow-up after diagnosis (maximum 12 months) was the number of months until the patient died or lost Medicare coverage. If neither of these events occurred before the end of the planned follow-up time after diagnosis, the patient was followed for the full 12 months. Non-cancer patients were frequency-matched by age to cancer patients at a ratio of 1:1; and coverage and follow-up requirements were the same as for STS cases. VTEs of interest were deep vein thrombosis (DVT), pulmonary embolism (PE), and other thromboembolic events (OTE). Thromboembolic events included in the OTE category were as follows: central retinal vein occlusion, venous tributary (branch) occlusion, nonpyogenic thrombosis of intracranial venous sinus, phlebitis/thrombophlebitis of superficial veins of lower and upper extremities, phlebitis/thrombophlebitis of other sites, gout with other specified manifestations, Budd–Chiari syndrome, venous embolism/thrombosis of renal vein, and portal vein thrombosis. ICD-9 diagnosis codes were used to identify VTEs and ICD-O-3 codes were used to identify STS patients (overall and by subtype). Types and definitions of STS by ICD-O-3 histology codes are as follows: GIST (8935, 8936), leiomyosarcoma (8890–91, 8894–97), malignant fibrous histiocytoma (8830), liposarcoma (8850–8855, 8858), dermatofibrosarcoma (8832, 8833), rhabdomyosarcoma (8900–8902, 8910, 8912, 8920), angiosarcoma (9120, 9130, 9133, 9170), nerve sheath tumor (9540, 9560–62), fibrosarcoma (8810, 8811, 8814, 8815), sarcoma NOS (not otherwise specified) (8800–8805), Ewing Sarcoma/primitive neuroectodermal tumor (9260, 9364, 9365, 9473), extraskeletal osteosarcoma (9180, 9181), extraskeletal chondrosarcoma (9220, 9231, 9240), synovial sarcoma (9040–9043), clear cell sarcoma (9044), myxosarcoma (8840), malignant hemangiopericytoma (9150), malignant giant cell tumor (9251, 9252), malignant granular cell tumor (9580), alveolar soft part sarcoma (9581), and desmoplastic small round cell tumor (8806). Bone and joint sites were excluded for all above mentioned diagnoses as were non-malignant tumor types. DVT was captured using ICD-9 codes of 451.1 (451.11, 451.19) 451.2, 451.81, 451.83, 451.84, 453.1, 453.2, 453.4 (453.40, 453.41, 453.42) 453.8, and 453.9; PE was captured using ICD-9 codes of 415.1 and 415.19; and OTEs were captured using ICD-9 codes of 362.35, 362.36, 437.6, 451.0, 451.82, 451.89, 451.9, 453.0, 453.3, and 452.

### Statistical analysis

#### STS patients

Incidence rates of each VTE (a) in the 12 months before diagnosis and (b) in the 12 months after diagnosis were described by age, race, sex, stage at diagnosis, and year of diagnosis. The numerator is the number of events that occurred over the respective 12-month period and the denominator was the person-years at risk. Events in the 12 months after diagnosis were further described as the proportion of cases with a first event in discrete time intervals of follow-up time (0–90 days, 91–180 days, 181–270 days, and 271–365 days). The numerator of each incidence proportion is the number of persons with their first event of interest during that time period only, while the denominator represents the persons who were alive and free of events at the beginning of the period.

Relative incidence rates of VTEs in STS patients with a recent history of cardiovascular event (CV) or VTE event (12 months before diagnosis) versus STS patients without such a recent history were calculated using the Cox proportional hazard models. Using a commonly used definition present in the literature, history of CV event was defined as any of the following events in the 12 months before STS diagnosis: myocardial infarction, ischemic stroke, congestive heart failure, angina, or TIA in the prior 12 months. The first VTE outcome was counted for each patient from time of diagnosis up to 12 months after diagnosis. Potential confounders, identified through ICD-9 diagnosis and procedure codes, were as follows: age at diagnosis, race, sex, diabetes, hypercholesterolemia, atherosclerosis, varicose veins, recent high-risk surgical procedure, central venous catheter, kidney disease, stage at diagnosis, surgery of primary tumor, chemotherapy, history of cancer. Results are presented by major STS subtypes [angiosarcoma, fibrosarcoma, GIST, leiomyosarcoma, liposarcoma, malignant fibrous histiosarcoma (MFH), nerve sheath tumor, and sarcoma NOS] where cell sizes permit.

The Cox proportional hazard model was used to build predictive models to identify important risk factors for each VTE of interest among STS patients. Potential risk factors included in the initial (full) model were as follows: age at diagnosis, race, sex, diabetes, hypercholesterolemia, atherosclerosis, varicose veins, recent high-risk surgical procedure, central venous catheter, kidney disease, stage at diagnosis, chemotherapy, hormone therapy, surgery of primary tumor, history of cancer, and recent history of VTE of interest. After stepwise backwards elimination, risk factors with a multivariable *p* value <0.1 were retained in the final multivariable predictive model.

#### STS vs. non-cancer patients

A matched-cohort design was utilized to evaluate the relative incidence rate of VTEs in STS patients (12 months before diagnosis) versus non-cancer cases (12 months before index date). Multivariable logistic regression modeling was performed. Potential confounders assessed were as follows: race, sex, diabetes, hypercholesterolemia, atherosclerosis, varicose veins, recent high-risk surgical procedure, central venous catheter, kidney disease, recent history of cardiovascular or VTEs. All models were adjusted for age to account for the age-matched design.

Relative incidence rate of VTEs in cancer patients (12 months after diagnosis) versus non-cancer cases (12 months after index date) was calculated using multivariable Cox proportional hazard models. Matching was accounted for by including the matching variable (age) in the STRATA statement. Potential confounders assessed were the same as in aforementioned logistic regression models. Where possible, results are presented by major STS subtypes [angiosarcoma, fibrosarcoma, GIST, leiomyosarcoma, liposarcoma, malignant fibrous histiosarcoma (MFH), nerve sheath tumor, sarcoma NOS]. Sas 9.1 was used to perform all analyses.

## Results

The study population for the first series of analyses consisted of 3,480 STS patients 65 years of age and older (median age = 77). Eighty-five percent of the population was white, 9% of the population was black, and 6% was another race. Forty-seven percent of STS patients were male. The distribution of cases by stage was as follows: 43% localized, 23% regional, 21% distant, and 14% unstaged. The most common STS subtypes in our data were sarcoma NOS (40.9%), GIST (24.3%), leiomyosarcoma (7.6%), MFH (6.4%), and angiosarcoma (5.3%) (Table 1). The two most common primary sites were connective or subcutaneous tissue (35.0%) and the digestive system (31.8%) (Table 1). The non-cancer comparison cohort (n = 3,480) was similar in its distribution of age, race, and sex (Table 1).

**Table 1:** SEER-Medicare study population (1993–2005): soft tissue sarcoma patients (n = 3,480) and non-cancer controls (n = 3,480)

	Cancer		Non-cancer	
	n	%	N	%
Age in years				
65–69	475	13.7	549	15.8
70–74	825	23.7	836	24.0
75–79	880	25.3	853	24.5
80–84	744	21.4	715	20.6
85+	556	16.0	527	15.1
Race				
Black	300	8.6	301	8.7
White	2,967	85.3	2,945	84.6
Other/unknown race	213	6.1	234	6.7
Sex				
Female	1,843	53.0	1,972	56.7
Male	1,637	47.0	1,508	43.3
STS subtype				
Sarcoma NOS	1,422	40.9	na	na
GIST	845	24.3	na	na
Leiomyosarcoma	264	7.6	na	na
Maligant fibrous histiocytoma	224	6.4	na	na
Angiosarcoma	184	5.3	na	na
Liposaroma	136	3.9	na	na
Nerve sheath tumor	118	3.4	na	na
Fibrosarcoma	64	1.8	na	na
Synovial Sarcoma	49	1.4	na	na
Osteosarcoma	44	1.3	na	na
Rhabdomyosarcoma	25	0.7	na	na
Chondrosarcoma	25	0.7	na	na
Dermatofibrosaroma	22	0.6	na	na
Ewing sarcoma	19	0.5	na	na
Maligant hemangiopericytoma	15	0.4	na	na
Clear cell sarcoma	<11	0.3	na	na
Myxosarcoma	<11	0.2	na	na
Alveolar soft part	<11	0.1	na	na
Maligant granular cell tumor	<11	0.1	na	na
Maligant giant cell tumor	<11	0.0	na	na
Primary site				
Connective/subcutaneous tissue	1,217	35.0	na	na
Digestive system	1,107	31.8	na	na
Respiratory system	279	8.0	na	na
Breast	178	7.7	na	na
Other non-epithelial skin	167	5.1	na	na
Female genital system	160	4.8	na	na
Urinary system	105	4.6	na	na
Other and unknown	267	3.0	na	na
Stage				
Unstaged	482	13.9	na	na
Localized	1,482	42.6	na	na
Regional	800	23.0	na	na
Distant	716	20.5	na	na

Per SEER Medicare data use agreement, cell sizes <11 cannot be displayed.

*na* not applicable.

### STS patients

Among STS patients, DVTs occurred at the highest rate (149/1,000 person-years) of all VTEs after diagnosis (Table 2). The unadjusted incidence rate of VTEs was 1.7–4.1 times higher during the 12-month period after STS diagnosis than the 12-month period prior to cancer diagnosis (Table 2). Regardless of VTE type, over half of VTEs occurred in the first 90 days after STS diagnosis: DVT: 62% (228/367), PE: 67% (70/105), and OTE: 51% (55/108). This pattern did not vary by STS subtype (data not shown).

**Table 2:** Unadjusted incidence rates of venous thromboembolic events, before and after STS diagnosis

n = 3,480 STS^a^ patients	Incidence 12 months before STS diagnosis	Incidence 12 months after STS diagnosis
DVT^a^		
n/person-years^b^	137/3,418	367/2,468
Rate/1,000^c^	40.1 (33.7–164.7)	148.7 (133.9–164.7)
*Rate ratio* (*after vs. before*)^d^	–	*3.7* (*3.0*–*4.5*)
PE^a^		
n/person-years^b^	34/3,463	105/2,589
Rate/1,000^c^	9.8 (6.8–49.1)	40.6 (33.2–49.1)
*Rate ratio* (*after vs. before*)^*d*^	–	*4.1* (*2.8*–*6.1*)
OTE^a^		
n/person-years^b^	82/3,438	106/2,590
Rate/1,000^c^	23.9 (19.0–49.5)	40.9 (33.5–49.5)
*Rate ratio* (*after vs. before*)^d^	–	*1.7* (*1.3*–*2.3*)

Rate ratios are highlighted in italics.

^a^ *VTE* venous thromboembolic events, *STS* soft tissue sarcoma, *DVT* deep vein thrombosis, *PE* pulmonary embolism, *OTE* other thromboembolic event. OTE category includes the following diagnoses: central retinal vein occlusion, venous tributary (branch) occlusion, nonpyogenic thrombosis of intracranial venous sinus, phlebitis/thrombophlebitis of superficial vessels of lower extremities, phlebitis/thrombophlebitis of superficial veins of upper extremities, phlebitis/thrombophlebitis of other sites, gout with other specified manifestations, Budd–Chiari syndrome, and venous embolism/thrombosis of renal vein.

^b^ *n* number of VTE events, *p-y* person-years.

^c^Rates are per 1,000 person-years and are unadjusted. Age adjustment is unnecessary as these rates are intentionally representative of the older subpopulation (ages 65+) of STS patients. Only first VTE counted in rate estimates.

^d^Rate ratios are unadjusted.

When STS cases were stratified further by subtype, the highest rates of DVT and PE were seen in sarcoma NOS patients (DVT: 204/1,000 p-y and PE: 50/1,000 p-y). Rates of OTEs ranged from 18/1,000 to 50/1,000; however, estimates for most subtypes were based on very small numbers (Table 3). Unadjusted analyses revealed that STS patients with a recent history of a VTE had substantially higher rates of that specific VTE after STS diagnosis than those without history of that VTE (Table 3). STS patients with a recent history of a CVD event had slightly higher rates of VTEs after STS diagnosis than those without history of a CVD event. Patients of advanced or regional STS stage had higher rates of VTEs after diagnosis compared to patients with localized disease. Patients who were treated with chemotherapy were also more likely compared to patients without chemotherapy to experience a VTE after STS diagnosis. Patients who were treated with surgery of their primary tumor were half as likely as patients without surgery to experience a VTE after STS diagnosis.

**Table 3:** Unadjusted incidence rates of venous thromboembolic events after cancer diagnosis among older STS patients

	DVT^a^		PE^a^		OTE^a^	
	N/P-Y^b^	Rate/1,000^c^ (95% CI)	N/P-Y^b^	Rate/1,000^c^ (95% CI)	N/P-Y^b^	Rate/1,000^c^ (95% CI)
By major STS subtype						
Angiosarcoma (n = 184)	14/147	95.4 (52.1, 160.0)	<11	26.5 (7.2, 68.0)	<11	39.8 (14.6, 86.6)
Fibrosarcoma (n = 64)	<11	72.1 (19.6, 184.5)	0	0	<11	17.6 (0.4, 97.8)
GIST (n = 845)	94/683	137.6 (111.2, 168.4)	33/721	45.7 (31.5, 64.2)	23/730	31.5 (20.0, 47.3)
Leiomyosarcoma (n = 264)	19/210	90.6 (54.5, 141.4)	<11	28.0 (10.3, 60.8)	<11	32.6 (13.1, 67.1)
Liposarcoma (n = 224)	13/118	110.1 (58.6, 188.2)	<11	24.5 (5.1, 71.7)	<11	24.5 (5.0, 71.5)
MFH (n = 184)	20/178	112.2 (68.5, 173.3)	<11	21.9 (6.0, 55.9)	<11	27.3 (8.9, 63.6)
Nerve sheath tumor (n = 118)	15/93	160.8 (90.0, 265.3)	<11	40.7 (11.1, 104.2)	<11	49.8 (16.2, 116.3)
Sarcoma, NOS (n = 1,422)	166/812	204.4 (174.5, 237.9)	41/862	47.5 (34.1, 64.5)	41/858	47.8 (34.3, 64.8)
By history of VTE^e^						
Yes	61/51	1199.2 (917.3–1540.5)	11/14	785.5 (392.3–1406.0)	30/50	599.2 (404.3–855.4)
No	306/2,417	126.6 (112.8–141.6)	94/2,575	27.6 (24.3–31.1)	76/2,540	29.9 (23.6–37.5)
*Rate ratio* (*yes vs. no*)^d^	–	*9.5* (*7.2*–*12.5*)	–	*21.5* (*11.5*–*40.2*)	–	*20.0* (*13.1*–*30.6*)
By history of CVD event^f^						
Yes	120/579	207.3 (171.9–247.9)	29/623	46.6 (31.2–66.9)	39/619	63.1 (44.8–86.2)
No	247/1,889	130.8 (115.0–148.1)	76/1,966	38.7 (30.5–48.4)	67/1,971	34.0 (26.3–43.2)
*Rate ratio* (*yes vs. no*)^d^	–	*1.6* (*1.3*–*2.0*)		*1.2* (*0.8*–*1.8*)		*1.9* (*1.3*–*2.8*)
By disease stage						
Localized	133/1,236	107.6 (90.1–127.5)	38/1,290	29.5 (20.9–40.4)	34/1,295	26.3 (18.2–36.7)
Regional	93/586	158.7 (128.1–194.4)	26/614	42.3 (27.7–62.1)	34/610	55.7 (38.6–77.9)
Distant	86/380	238.9 (191.1–295.0)	25/385	65.0 (42.1–95.9)	19/387	49.1 (29.6–76.7)
*Rate ratio* (*regional and distant. vs. local*)^d^	–	*1.5* (*1.1*–*1.9*)	–	*1.4* (*0.9*–*2.4*)	–	*2.1* (*1.3*–*3.4*)
By chemotherapy						
Yes	74/324	227.9 (178.9–286.1)	20/348	57.5 (35.1–88.7)	19/346	54.9 (33.0–85.7)
No	293/2,413	136.7 (121.5–153.3)	85/2,241	37.9 (30.3–46.9)	87/2,244	38.8 (31.1–47.8)
*Rate ratio* (*yes vs. no*)^d^	–	*1.7* (*1.3*–*2.2*)	–	*1.5* (*0.9*–*2.5*)		*1.4* (*0.9*–*2.3*)
By surgery of primary tumor						
Yes	247/1,976	125.0 (109.9–141.6)	72/2,065	34.9 (27.3–43.9)	76/2,064	36.8 (29.0–46.1)
No	115/467	246.4 (203.5–295.8)	32/498	64.2 (43.9–90.7)	29/501	57.9 (38.8–83.1)
*Rate ratio* (*yes vs. no*)^d^	–	*0.5* (*0.4*–*0.6*)	–	*0.5* (*0.4*–*0.8*)	–	*0.6* (*0.4*–*1.0*)
By Primary site						
Connective/subcutaneous	136/830	163.9 (137.5–193.8)	33/877	37.6 (25.9–52.8)	37/875	42.3 (29.8–58.3)
Digestive system	120/832	144.2 (119.5–172.4)	38/879	43.2 (30.6–59.4)	29/885	32.8 (22.0–47.1)
Other/unknown^g^	111/805	137.8 (113.3–165.9)	34/833	40.8 (28.3–57.0)	40/830	48.2 (34.4–65.7)

Per SEER-Medicare data use agreement, cell sizes <11 cannot be displayed, nor can estimates that could be used to derive the undisplayed numbers <11. Rate ratios are highlighted in italics.

^a^ *DVT* deep vein thrombosis, *PE* pulmonary embolism, *OTE* other thromboembolic event. OTE category includes the following diagnoses: central retinal vein occlusion, venous tributary (branch) occlusion, nonpyogenic thrombosis of intracranial venous sinus, phlebitis/thrombophlebitis of superficial vessels of lower extremities, phlebitis/thrombophlebitis of superficial veins of upper extremities, phlebitis/thrombophlebitis of other sites, gout with other specified manifestations, Budd–Chiari syndrome, and venous embolism/thrombosis of renal vein.

^b^ *N* number of VTE events, *P-Y* person-years.

^c^Rates are per 1,000 person-years and are unadjusted. Age adjustment is unnecessary as these rates are intentionally representative of the older subpopulation (≥65 years) of STS patients. Only first VTE counted in rate estimates.

^d^Rate ratios are unadjusted.

^e^History of VTE of interest in the 12 months before STS diagnosis.

^f^History of CVD is defined as a history of any of the following events in the 12 months before STS diagnosis: myocardial infarction, ischemic stroke, onset congestive heart failure, angina, or TIA.

^g^Other/unknown category includes primary sites of the breast, female genital system, other non-epithelial, respiratory system, urinary system, other sites and unknown sites.

Multivariable modeling was conducted to more closely evaluate the association between VTE history and incidence of VTEs after sarcoma diagnosis by STS subtype. Overall, history of a VTE in the 12 months before sarcoma diagnosis substantially increased the risk of a subsequent VTE in the 12 months after STS diagnosis (HR range = 6.4–49.3) (Table 4). Although all age-adjusted associations were strong, the most marked associations were seen in patients with leiomyosarcoma (HR = 49.3, 95% CI = 22.2–109.0), GIST (HR = 20.9, 95% CI = 13.6–32.2), and liposarcoma (HR = 19.1, 95% CI = 4.2–87.4) (Table 4).

**Table 4:** Relative risk of VTE after STS diagnosis, recent versus no recent VTE history

STS subtype^a^	Hazard ratio	95% confidence interval
Angiosarcoma	6.4	(1.9–21.8)^+^
GIST	20.9	(13.6–32.2)^+^
Leiomyosarcoma	49.3	(22.2–109.0)^+^
Liposarcoma	19.1	(4.2–87.4)^+^
MFH^b^	11.9	(3.6–39.4)^+^
NST^c^	15.8	(5.8–42.7)^+^
Sarcoma NOS^d^	15.4	(10.9–21.8)^+^

All models adjusted for age. Recent VTE history = history of VTE in the 12 months before STS diagnosis. *VTE* venous thromboembolic event, including deep vein thrombosis, pulmonary embolism, and other thromboembolic events. The other thromboembolic event category includes the following diagnoses: central retinal vein occlusion, venous tributary (branch) occlusion, nonpyogenic thrombosis of intracranial venous sinus, phlebitis/thrombophlebitis of superficial vessels of lower extremities, phlebitis/thrombophlebitis of superficial veins of upper extremities, phlebitis/thrombophlebitis of other sites, gout with other specified manifestations, Budd–Chiari syndrome, and venous embolism/thrombosis of renal vein.

^a^Hazard ratios for STS subtypes other than those listed were not estimable due to small numbers.

^b^Maligant fibrous histiocytoma.

^c^Nerve sheath tumor.

^d^Sarcoma not otherwise specified.

^+^Statistically significant.

Predictors of each VTE subtype after sarcoma diagnosis were evaluated among the combined group of STS patients. There were only two statistically significant predictors of increased risk of PE after STS diagnosis: atherosclerosis and recent history of PE. There were four predictors of increased risk of OTE after STS diagnosis: atherosclerosis, hormone therapy, recent history of OTE and varicose veins. There were multiple predictors of both increased risk and decreased risk of DVT among the combined group of STS patients. Predictors of increased risk of DVT included kidney disease, radiation treatment, regional or distant stage and varicose veins; predictors of decreased risk of DVT included surgery of primary, high risk surgery, and central venous catheter. By far, the strongest predictor of all VTE events after STS diagnosis was a history of that same event in the 12 months before diagnosis (DVT: HR = 7.6, 95% CI = 5.7–10.1; PE: HR = 17.6, 95% CI = 9.4–33.0; OTE: HR = 16.6, 95% CI = 10.8–25.4) (data not shown). Presence of atherosclerosis was also an important predictor for all VTE events, associated with a 1.8–2.5 times greater risk of VTE (DVT: HR = 1.8, 95% CI = 1.4–2.3; PE: HR = 2.0, 95% CI = 1.3–3.1; OTE: HR = 2.5, 95% CI = 1.7–3.8) (data not shown).

### STS patients compared to an age-matched, non-cancer population

Angiosarcoma and sarcoma NOS patients were more likely to have experienced a VTE in the recent past (i.e. 12 month prior to STS diagnosis/index date) than non-cancer individuals (Table 5). A strong association between STS and VTE after diagnosis (or index date for non-cancer patients) was observed in most STS subtypes. The strongest comparisons of VTE after diagnosis for cancer versus non-cancer patients were among angiosarcoma (HR = 9.1, 95% CI = 2.1–39.5), leiomyosarcoma (HR = 5.5, 95% CI = 2.1–15.0) and sarcoma NOS patients (HR = 5.2, 95% CI = 3.8–6.9) (Table 5).

**Table 5:** Relative risk of VTEs before and after STS diagnosis/index date: STS versus non-cancer cohort

STS subtype^a^	Before odds ratio (95% CI)	After hazard ratio (95% CI)
Angiosarcoma	4.0 (1.4, 11.1)^+^	9.1 (2.1, 39.5)^+^
Fibrosarcoma	0.5 (0.1, 2.0)	1.0 (0.1, 16.0)
GIST	1.0 (0.7, 1.4)	4.0 (2.8, 5.7)^+^
Leiomyosarcoma	1.5 (0.6, 3.5)	5.5 (2.1, 15.0)^+^
Liposarcoma	Not estimable	2.5 (0.9, 7.6)
MFH^b^	1.4 (0.6, 3.3)	19.7 (3.7, 106.0)^+^
NST^c^	2.1 (0.8, 5.4)	4.4 (1.1, 17.5)^+^
Sarcoma NOS^d^	1.9 (1.4, 2.6)^+^	5.2 (3.8, 6.9)^+^

All models adjusted for age at index/diagnosis date. Estimates represent the relative risk of VTE event in the 12 months before or after diagnosis/index date. *STS* *patients* 3,840; non-cancer patients = 3,480. *VTE* Venous Thromboembolic Event (including deep vein thrombosis,pulmonary embolism, and other thromboembolic events). OTE category includes the following diagnoses: central retinal vein occlusion, venous tributary (branch) occlusion, nonpyogenic thrombosis of intracranial venous sinus, phlebitis/thrombophlebitis of superficial vessels of lower extremities, phlebitis/thrombophlebitis of superficial veins of upper extremities, phlebitis/thrombophlebitis of other sites, gout with other specified manifestations, Budd-Chiari syndrome, and venous embolism/thrombosis of renal vein.

^a^Hazard ratios for STS subtypes other than those listed were not estimable due to small numbers.

^b^Maligant fibrous histiocytoma.

^c^Nerve sheath tumor.

^d^Sarcoma not otherwise specified.

^+^Statistically significant.

## Discussion

We studied the incidence of venous thromboembolic events before and after soft tissue sarcoma diagnosis. There are only four published studies, to our knowledge, that have investigated VTEs in adult STS patients. Two of these are large studies of thromboembolic events among cancer patients that include sarcomas as a cancer site [6, 7]. Khorana et al. [6] reported results from a retrospective cohort study (n = 1,597 sarcoma patients) including patients receiving chemotherapy at any one of 115 University HealthSystem Consortium locations between the years of 1995 and 2002. During their first hospitalization for neutropenia, 4.8% of sarcoma patients had a VTE. Khorana et al. [7] conducted a retrospective cohort study (n = 21,989 sarcoma cases) of the same health consortium and found that 2.9% of sarcoma patients experienced a VTE during one hospitalization following cancer diagnosis. Results from these two large studies are difficult to compare to our incidence proportions for several reasons: in these studies, bone sarcomas were included in their sarcoma category, median age of the sarcoma group was not stated, and incidence proportions were based on a VTE diagnosis during a single hospitalization. Our analysis evaluated incidence proportions and incidence rates (Table 2) over the full 12-month period (or death) after diagnosis.

Two studies reported incidence of venous thromboembolic events (VTEs) among soft tissue sarcoma patients, specifically [14, 15]. Mitchell et al. [15] conducted a retrospective cohort study of VTE events among trunk/extremity soft tissue or bone sarcoma patients presenting to their unit between 1998 and 2003. Among STS patients (n = 158), 5.0% experienced a DVT and 1.3% experienced a PE after diagnosis. Damron et al. [14] conducted a retrospective cohort study of patients receiving surgery for bone and soft tissue sarcoma of the head, neck, upper extremity and lower extremity. There were 120 STS cases included and 4.2% of those cases experienced one or more VTE following surgery (2.5% DVT and 2.5% PE). Incidence proportions from these two studies were lower than what we found in our study (10.6% DVT and 3.0% PE). Both of these studies, however, were very small and had much younger patient populations than ours.

Our study found that STS patients undergoing chemotherapy were roughly 50% more likely (DVT: RR = 1.7, PE: RR = 1.5, OTE: RR = 1.4) to experience a DVT, PE or OTE after diagnosis, although only about 10% of our STS patients received chemotherapy treatment (Table 3). Damron et al. [14] reported a similar result; STS patients undergoing chemotherapy treatment were at an increased risk of VTE compared to those not receiving chemotherapy (p = 0.04).

An important component of our analysis was the evaluation of VTE history as a risk factor for future VTE events. Our results suggest that VTE history is the most important factor to consider in evaluating risk of future VTE in STS patients. There are no other studies in the current literature that quantify the association between VTE history and future events among STS patients; however, this result is consistent with broader studies of VTE among cancer patients [16, 17].

The major risk factors for VTEs among cancer patients reported in the literature are increased age, female sex, African American race, renal disease, infection, pulmonary disease, obesity, arterial thromboembolism, inherited prothrombotic mutations, prior history of VTE, performance status, advanced stage cancer, major surgery, hospitalization, chemotherapy, hormone therapy, anti-angiogenic agents, erythropoiesis-stimulating agents, transfusions, and central venous catheters [7, 12, 18]. In our predictive models, many of these risk factors proved to be predictors of VTEs in the 12 months after STS diagnosis.

A few interesting differences are worth discussion. Atherosclerosis was a strong predictor for DVT, PE and OTE events in our data. This condition is not generally mentioned as a risk factor for VTE among cancer patients; however, cardiovascular literature has suggested a link between these two conditions [19–22]. We also observed that central venous catheterization, high-risk surgery, and surgery of primary were associated with a decreased risk of DVT after STS diagnosis. Decreased risk in this subgroup of patients is likely due to the close monitoring and prophylactic treatment for venous thromboses in surgical and catheterization situations.

Finally, our analysis compared the risk of VTE events in STS versus non-cancer patients both before and after STS diagnosis. Our study found that patients with angiosarcoma and sarcoma NOS were more likely to have experienced a VTE in the recent past (i.e. 12 month before STS diagnosis) than non-cancer individuals. These results support the theory that VTE could be a risk marker for an ensuing STS diagnosis or, perhaps, a misdiagnosis. Several authors of case studies suggested that STSs may present as or be mistaken for a VTE, underlining the importance of investigating potential tumors when diagnosing VTE. In particular, STSs of the lower extremities have been misdiagnosed as DVTs [23–26] and pulmonary sarcomas/leiomyosarcomas have been misdiagnosed as pulmonary embolisms [27–40].

There are several strengths of note for this study. To our knowledge, this is the first study to examine VTEs among older STS patients. In this analysis we were able to focus on STS patients in particular, rather than the broader category of sarcoma which includes bone sarcomas. The STS patient cohort was large (n = 3,840) allowing us to provide data by STS subtype. The wealth of the data in the SEER-Medicare database allowed us to quantify the occurrence of TE events before STS diagnosis and during various time periods after STS diagnosis, and to make comparisons between STS patients and age-matched non-cancer individuals. Furthermore, we were able to adjust for and/or stratify by important covariates in our analysis. No previous studies performed multivariate analyses on STS patients nor did they investigate the timing of VTE events among STS patients.

As in any study, limitations were present. Many effect estimates are imprecise, present with wide confidence intervals, and should be interpreted cautiously with the direction of the effect emphasized over the magnitude. The STS classification of MFH was changed in 2002 because the large majority of tumors formerly classified as MFH can be more meaningfully classified as other tumor types. However, due to the timeframe of SEER-Medicare data collection, the old categorization had to be used for this study. The results based on this older cohort (i.e., 65 years or older) are generalizable only to those of the same age group. Also, information on some behavioral risk factors such as smoking, sedentary lifestyle, immobility, and CVD family history was unavailable. Finally, we had no access to information about potential predictive biomarkers such as elevated platelet or leukocyte counts, tissue factor, soluble p-Selectin, D-dimer, factor V Leiden, and prothrombin 20210A mutations [12, 41, 42].

## Conclusion

This is the first study to perform an in depth analysis of VTEs among older STS patients. Our results indicate that STS patients are at increased risk of VTEs after cancer diagnosis and that patients with a history of VTE are much more likely to have a subsequent VTE in the 12 months after their sarcoma diagnosis. VTEs are common and serious co-morbidities that should be closely monitored in older STS patients, particularly during the first 3 months after diagnosis and among those with a recent history of a VTE.
